# Microfluidic Flows and Heat Transfer and Their Influence on Optical Modes in Microstructure Fibers

**DOI:** 10.3390/ma7117566

**Published:** 2014-11-24

**Authors:** Edward Davies, Paul Christodoulides, George Florides, Kyriacos Kalli

**Affiliations:** 1 Nanophotonics Research Laboratory, Department of Electrical Engineering/Computer Engineering and Informatics, Cyprus University of Technology, 3603 Limassol, Cyprus; E-Mail: edd_dave@msn.com; 2 Faculty of Engineering and Technology, Cyprus University of Technology, 3603 Limassol, Cyprus; E-Mails: paul.christodoulides@cut.ac.cy (P.C.); gflorides@cablenet.com.cy (G.F.)

**Keywords:** microstructure optical fibers, heat transfer, microfluidics

## Abstract

A finite element analysis (FEA) model has been constructed to predict the thermo-fluidic and optical properties of a microstructure optical fiber (MOF) accounting for changes in external temperature, input water velocity and optical fiber geometry. Modeling a water laminar flow within a water channel has shown that the steady-state temperature is dependent on the water channel radius while independent of the input velocity. There is a critical channel radius below which the steady-state temperature of the water channel is constant, while above, the temperature decreases. However, the distance required to reach steady state within the water channel is dependent on both the input velocity and the channel radius. The MOF has been found capable of supporting multiple modes. Despite the large thermo-optic coefficient of water, the bound modes’ response to temperature was dominated by the thermo-optic coefficient of glass. This is attributed to the majority of the light being confined within the glass, which increased with increasing external temperature due to a larger difference in the refractive index between the glass core and the water channel.

## 1. Introduction

Microfluidics is a multidisciplinary field concerned with the design and application of the transport of small volumes of fluids, typically of the order of nano- to femto-liters. A primary goal using this technology is the control and sorting of nano-particles. To date, the most successful commercial application of microfluidics is the inkjet printer; however, it has also been used as biochips to integrate assay operations, such as detection and pre-treatment, for enzymatic analysis, DNA analysis and proteomics. Optical fibers have long been used as biological sensors, offering high levels of sensitivity and a biologically compatible reaction surface, typically glass. Microstructure optical fibers guide light through either a difference in refractive index or by the formation of a bandgap. Typically, MOFs consist of a waveguide(s) surrounded by a series of holes, which have radii of the order of a micrometers. This makes them of particular interest, whereby a fluid, transported in the holes surrounding the core can interact with the guided mode of the fiber [1,2,3,4]. More recently, there has been a move towards microfluidic fiber microreactors (as flow-through systems) [5].

Microstructure fibers are excellent candidates as microfluidic devices, given the minute sample volumes that are required and as optical fiber sensors are well suited to applications in chemical, biological and medical sensing [6,7,8,9,10,11]. Furthermore, MOFs have found uses in non-linear switching and laser emission devices, where optofluidics is critical [12,13,14]. Given the development of new types of MOFs with cross-sections containing circular, or elliptical holes, or more complex cross-sectional geometries, it is important to be able to model the fluid transport capabilities of these fiber types. This becomes ever more important as advanced infusion and infiltration methods are realized. Furthermore, complex cross-sectional geometries can affect the transfer of heat into the fiber, creating local changes in the behavior of the fluid system.

To date, several studies on micro-fluidic flows have been undertaken. Yanhg *et al.* fabricated microfluidic devices with multiple channels of trapezoidal and triangular cross-sections and investigated the fluid dynamics of the devices as a function of the flow rate, the channel depth and the channel’s geometry [15]. Colin and Tancogne studied the stability of jets in micro-channels and computed the length for which a jet is stable for a given configuration with respect to the flow rates, viscosities, diameter of the channel and surface tension [16], whereas Sahu *et al.* studied the stability of a co-flow composed of a Newtonian and non-Newtonian fluid [17]. Lien and Vollmer detected minimum flow rates based on integrated optical fiber cantilevers [18]. Regarding heat transfer related to microfluidics, the literature includes the work of Beskok and Karniadakis, who performed simulations of heat and momentum transfer in micro-geometries [19], Chen and Wu’s study of the thermal properties of a micro-channel flow in miniature thermal conductivity detectors [20] and Damean *et al.*’s study of the fluid and flow characteristics through a one-dimensional model for the heat transfer in a micro-electromechanical system for microfluidics [21]. However, one cannot assume that the flow dynamics in MOFs will be the same as conventional micro-fluidic channels, where the length scales are a factor of 10 or more greater.

**Figure 1:**
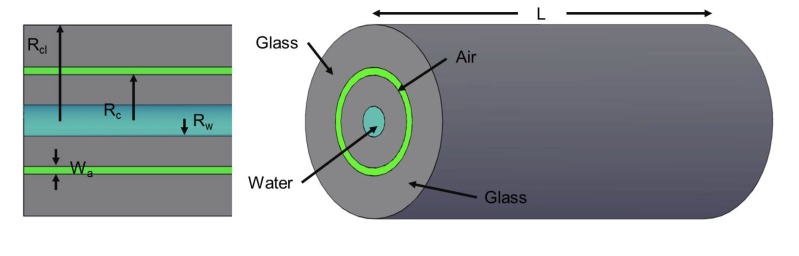
Schematic of the MOF used in the modeling, including labels for the materials and geometries.

The effect of temperature and fluid flow on the optical properties of a MOF has yet to be considered; therefore, it is necessary to undertake a study of how MOFs behave as microfluidic channels and optical waveguides, concurrently. In this paper, finite element analysis (FEA) is used with the Navier–Stokes equations and the convection diffusion equations to investigate the influence of flow rates, fiber geometry and external temperature on a MOF. A schematic of the MOF studied is shown in Figure 1 and was chosen, since it built and expanded upon the work of Christodoulides *et al.* [22]. Considering the optical properties, using the steady-state region of the MOF, the temperature change is converted into an equivalent change in refractive index, using the appropriate thermo-optic coefficients, where FEA is employed to calculate the bound modes of the MOF and their effective indices using the Helmholtz equation.

The remainder of this paper is organized as follows. Section 2 describes the theory used for the numerical analysis. Section 3 outlines the parameters and boundary conditions used in writing the FEA model. Section 4 eports the fluidic, thermal and optical results gathered from the FEA model and a discussion. Conclusions are contained in Section 5.

## 2. Theory

This section outlines the basic theory that the FEA analysis employs in the MOF investigation. For the ease of exposition, the theory is divided into three sections. The first section describes laminar flow, the second heat transfer for solids and fluids and the final section the optical mode analysis. For convenience, Table 1 is included here with all of the symbols. We are only concerned with the optical response when the MOFs are at steady state, and due to the small mass of the MOF, the dynamic response is much less than 1 s. As a consequence, only the time-independent solutions are considered here. Concerning the modeling of heat transfer, only convective and conductive heat transport mechanisms are considered here, because, due to the small change in temperature, radiative losses are expected to be negligible.

**Table 1:** Parameters used in the theory and modeling of the MOFs.

Symbol	Description	Units	Symbol	Description	Units
*u*	Mean velocity	m s *^−^*^1^	*T*	Temperature	K
*k*	Thermal conductivity	W m*^−^*^1^ K*^−^*^1^	*Q*	Heat source	W m*^−^*^3^
*C_p_*	Specific heat capacity	J kg *^−^*^1^ K*^−^*^1^	*ρ*	Density	k gm*^−^*^3^
*Q_v__h_*	Viscous heating	W m*−*^3^	*W_v_*	Work pressure	J m*^−^*^3^
*p*	pressure	N m*^−^*^2^	*I*	identity matrix	-
*µ*	Viscosity	Pa.s	*F*	Other forces	N
*E*	Electric field	V m*^−^*^1^	*κ*_o_	Wave number	m*^−^*^1^
*n*	Refractive index	*−*	-	-	-

### 2.1. Laminar Flows

A laminar flow is when a fluid flows in parallel layers with no disruption between the layers and, at low velocities, with no lateral mixing. From the Navier–Stokes equations at steady state, the two equations governing the behavior in MOFs are:

(1) *∇.u* = 0

(2) *ρ*(*u.∇*)*u* = *∇{−pI* + *µ*(*∇u* + (*∇u*)*^T ^*)*}* + *F*

Equation (1) is the continuity equation and states that the flow is continuous; it has no breaks in it. Equation (2) is the momentum balance equation for an incompressible flow. The left-hand side term is convective acceleration, which is caused by a change in velocity over the position, e.g., the speeding up of a fluid entering a converging nozzle. The first term on the right-hand side is the pressure gradient, which says that pressure accelerates flow from high pressure regions to low pressure regions. The second term on the right-hand side takes into account the viscosity and how it relates to temperature and implies that viscosity operates as a diffusion of momentum (similar to the diffusion of heat in the heat equation). The last term accounts for any other force; however, this is typically zero, because for microfluidics, other forces, such as gravity, are considered negligible. For the governing equations, it is useful to define a dimensionless number, called the Reynolds number, which, for a pipe, is given by:

(3) $$R_{e}=\frac{2\rho uR}{µ}$$

The formula describes the balance between inertial force and viscosity, where *R* is the radius of the pipe. In a microfluidic channel, the flows in a pipe can be classified according to their Reynolds number: *R_e_* < 1 is a laminar flow with no lateral convection; 1 < *R_e_* < 2,300 is a “intermediate” laminar flow where lateral convection becomes increasingly important with increasing Reynolds number; and finally, *R_e_* > 2,300 is a turbulent flow where a laminar flow can no longer be sustained and the velocity vector over time is no longer predictable [23].

### 2.2. Heat Transfer in Solids

The steady-state equation governing heat transfer in a solid is shown in Equation (4).

(4) *ρC_p_u.∇T* = *∇*.(*k∇T*) + *Q*

The left-hand side term describes a moving heat source/temperature difference, and this accounts for the convective cooling of the flowing fluid in the MOF. The first term on the right-hand side is the basis of Fourier’s law and describes the transfer of heat through a solid: conduction. The second term on the right-hand side is a heat source.

### 2.3. Heat Transfer in Fluids

The steady-state equation governing heat transfer in a fluid is shown in Equation (5). Again, the left-hand term describes heat transfer via convection, while the first term on the right-hand side describes conduction. Either method of heat transfer can dominate, and this is determined by the geometry of study. *Q_vh_* is the contribution of viscous heating, heating from the friction of the fluid on the channel walls, and *W_p_* is the work pressure term.

(5) *ρC_p_u.∇T* = *∇*.(*k∇T*) + *Q* + *Q_vh_* + *W_p_*

### 2.4. Optical Mode Analysis

The governing equation for optical mode analysis is an eigenvalue equation derived from the Helmholtz equation, shown in Equation (6). Taking the cross-section of the fiber as the *x*-*y*-axis and the length of the fiber as the *z*-axis, the light propagates in the *z* direction. If only considering core modes, the light is confined within the core of the MOF, resulting in an amplitude that goes to zero as it approaches the outer edge. This is simulated in the model by setting the electric field to zero at the outer cladding surface.

(6) *∇ ×* (*∇ ×E*) + *κ*_o_^2^*n*^2^*E* = 0

## 3. FEA Modeling

### 3.1. Thermo-Fluidic

The properties of the MOF were studied by 3D FEA using Comsol Multiphysics software. A schematic of the MOF used in this study is shown in Figure 1. The fiber consists of a single central water channel surrounded by a glass core. This is surrounded by an array of microstructures to create a difference in the refractive index. However, in the model, this region is considered to be purely air for computational efficiency. Finally, there is an outer region consisting of solid glass. Concerning the fluid flow, all outer walls are modeled as being non-slip, which enforces the continuity of the temperature between the fluid and solid domains. At the front fiber surface, a water inlet with a prescribed input velocity is selected and fixed at *T_o_*. At the opposite fiber end, an outlet is prescribed with no resistance pressure and coupled with an outflow boundary condition (for convection-dominated heat transfer). For the remaining, boundaries at the ends of the fiber are set as open boundaries set at *T_o_*. For solids, this allows heat flux to flow into and out of the modeled region, while for liquids, it also allows the flow of fluids into and out of the modeled region. The advantage of such a boundary condition is that it greatly reduces the region to be modeled. Finally, the outer radial surface of the fiber was designated as an inward heat flux set at *T_ext _* with a heat transfer coefficient of 600 Wm*^−^*^2^K*^−^*^1^. This is to simulate a surrounding heating element with an air interface of ~40 µm and is taken from the work of Christodoulides *et al.* [24]. To establish the performance of the MOF fiber, a parameter sweep of *T_ext_*, *u* and *R_w_*was conducted, with values ranging from 30 °C to 90 °C, 0.001 ms*^−^*^1^ to 0.1 ms*^−^*^1^ and 5 µm to 20 µm, respectively. The velocity values were chosen, because they gave a Reynolds number that ensured an intermediate laminar flow and are consistent with the literature [25]. The geometry values used in modeling the MOF are shown in Table 2, while the material parameters are taken from the literature. Analyzing the data in a program written in MATLAB, the steady-state distance and temperature of the water channel were measured.

**Table 2:** MOF geometry parameters.

Name	Description	Value
*R_c_*	Radius of the core glass	30 µm
*R_w_*	Radius of the water channel	5–20 µm
*R_cl_*	Radius of the MOF	62.5 µm
*W_a_*	Width of the air channel	5 µm
L	Total length of the modeled MOF	3 cm

### 3.2. Optical Mode

In the steady-state region of the MOF, the temperature distribution results in the fiber geometry being ~*T_ext_* (*i.e.*, minimal variation between the external and internal temperatures). To model the effects this has on the fiber modes, a mode analysis program was written in Comsol, which employs Maxwell’s equations. Only electromagnetic (EM) modes in the glass core are considered here, because only these modes will interact with the water channel, where any analyte would be found. From this, the boundary condition of the outer diameter of the optical fiber is designated as a perfectly electric conductor, signifying the magnetic field being zero at the outer edges. To model the refractive index of the material, two effects have to be accounted for: dispersion and the thermo-optic effects. Thermal expansion of the material is assumed to be negligible, since its effects are at least an order of magnitude smaller than the thermo-optic effect. Firstly, the glass dispersion was accounted for using the Sellmeier equation for glass, while for water, experimentally determined values were tabulated and an interpolation method employed for intermediate values. Secondly, a thermo-optic term was added to the refractive index of each material, *∇T.dn/dT*, where the thermo-optic coefficient of glass and water is 8.6 *×* 10^6^ K*^−^*^1^ [26] and *−*96.5 *×* 10^6^ K*^−^*^1^ [27], respectively.

## 4. Results and Discussion

### 4.1. Thermo-Fluidic Modeling

**Figure 2:**
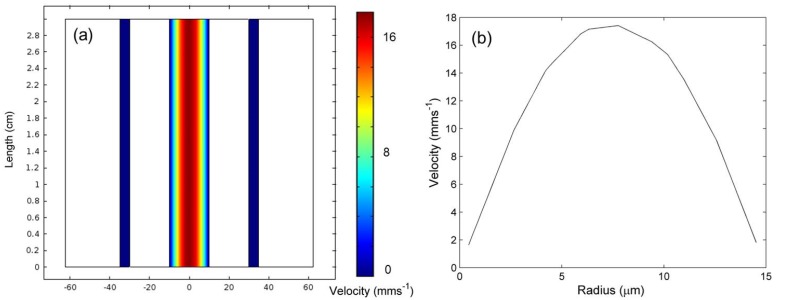
(**a**) The velocity cross-section for the entire MOF geometry and (**b**) the velocity profile with, expected, laminar flow.

Considering the fluid model predictions, in Figure 2, the velocity cross-section for the entire MOF geometry along with a velocity profile is shown, for a water channel radius of 10 µm and an input velocity of 0.01 ms*^−^*^1^. Figure 2a shows that the water channel has the largest velocity, but the air channel, surrounding the glass core, has a minimal velocity. This confirms that the effects of convection are being considered in the model, but its influence is small due to the negligible velocity observed. Figure 2a,b clearly shows a laminar fluid flow with the characteristic behavior of a parabolic velocity profile with an average velocity of ~ 0.01 ms*^−^*^1^, close to that of the input value and conserving velocity. These are typical results, and laminar flows were observed for all fiber geometries and velocities, which is expected considering the Reynolds numbers.

**Figure 3:**
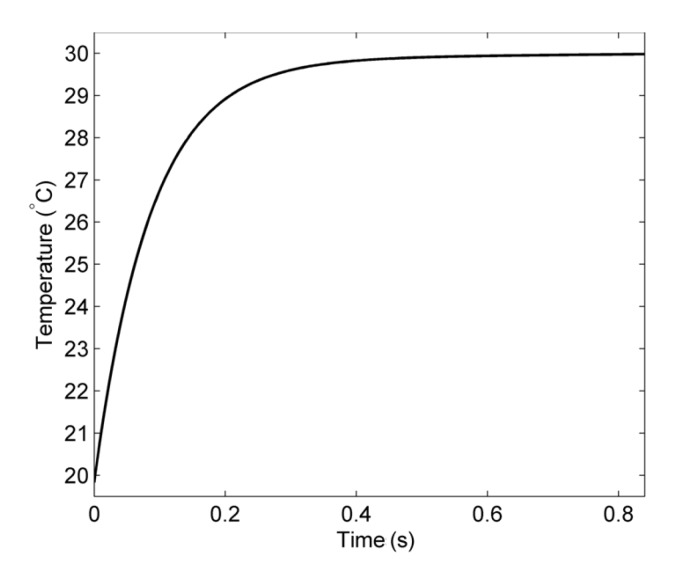
Time taken to reach the steady state within the MOF fiber by plotting the water channel temperature *vs*. time.

The small mass of the MOF results in a rapid response time to reach the steady state: less than 1 s. For most applications, this can be considered almost instantaneous. To confirm this, an investigation was conducted. The results are shown in Figure 3, where the steady-state temperature of the water channel is plotted *vs.* time. This shows that the time taken to reach thermal equilibrium within the water channel of the MOF is ~0.6 s and is consistent with the experimental work of Kalli *et al.* [28,29]. This confirms the response of the MOF, and as such, only steady-state solutions are considered further in this study.

**Figure 4:**
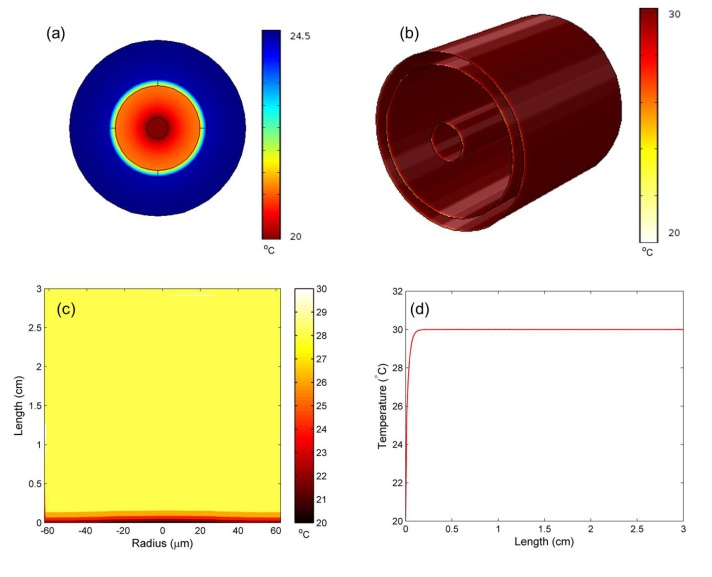
Typical steady-state results for the steady state of an MOF with a water channel radius of 10 µm, with an external temperature of 30 °C and an input velocity of 0.01 ms*^−^*^1^, with each label showing the (**a**) front fiber end surface temperature distribution; (**b**) 3D temperature distribution of the entire geometry; (**c**) cross-sectional temperature distribution and (**d**) temperature of the water channel *vs.* the length of the MOF.

Looking at the temperature, the modeled results at the steady state of an MOF with a water channel radius of 10 µm, with an external temperature of 30 °C and an input velocity of 0.01 ms*^−^*^1^ are shown in Figure 4. Figure 4a shows the input fiber end surface showing the cooling effect of the flowing water. Figure 4b shows the thermal profile of the entire MOF, while Figure 4c is a thermal distribution cross-section of the MOF geometry. Both figures show that, for these parameters, the cooling effect of the flowing water is limited to regions near the fiber end surface, with the majority of the MOF reaching the external temperature. Finally, Figure 4d is the temperature of the water channel *vs.* MOF length. This confirms that the water channel rapidly reaches the external temperature, within 1 mm.

**Figure 5:**
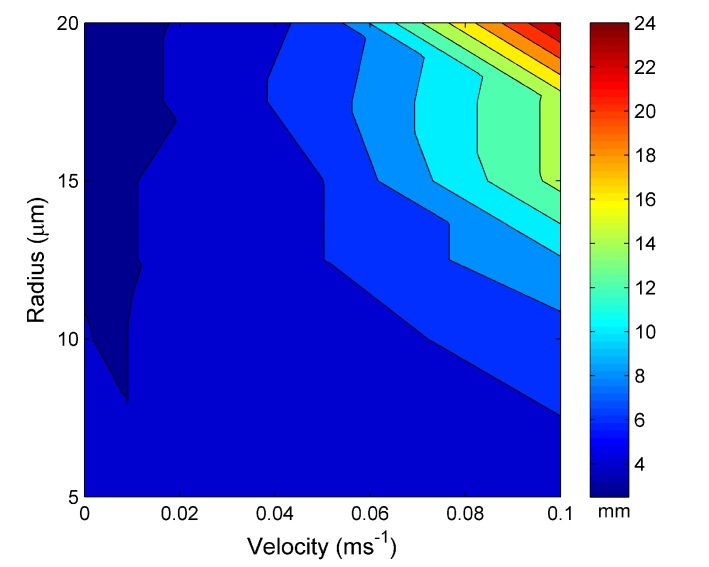
The distance taken to reach the steady-state length *vs.* the water hole radius and water input velocity at 30 °C.

Expanding upon this for multiple parameters, Figure 5 shows the distance required for the MOF to reach the steady-state temperature within the water channel *vs.* the input velocity and water channel radius for an external temperature set at 30 °C. There is a general trend of increasing steady-state distance with increasing water velocity and channel radius. For the parameters investigated here, there is a maximum of 24 µm at a velocity of 0.1 ms*^−^*^1^ and a water channel radius of 20 µm. This trend is expected, by increasing the radius or the velocity of the water cooling rate (the rate at which heat is removed from the modeled MOF fiber) of the optical fiber also increases and, thus, requires a greater distance for the surrounding heat source to have an equivalent effect. 

**Figure 6:**
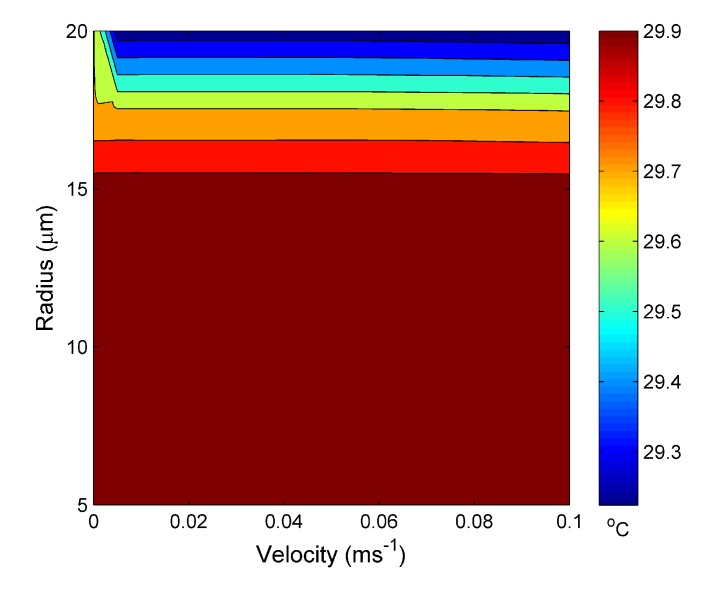
Calculated temperature at the steady state *vs.* the water hole radius and water input velocity at 30 °C.

Figure 6 shows the temperature reached at steady state for the water channel, again, *vs.* the input velocity and water channel radius for an external temperature set at 30 °C. The steady-state temperature is ~30 °C with only a ~0.6 °C variation between maximum and minimum temperatures. The plotted data also suggest that, except at very low velocities, the steady-state temperature is independent of water velocity, but dependent on the water channel radius. There is a critical channel radius of ~20 µm. Above this value, the steady state temperature decreases with increasing channel radius, while below this, the temperature is approximately constant.

**Figure 7:**
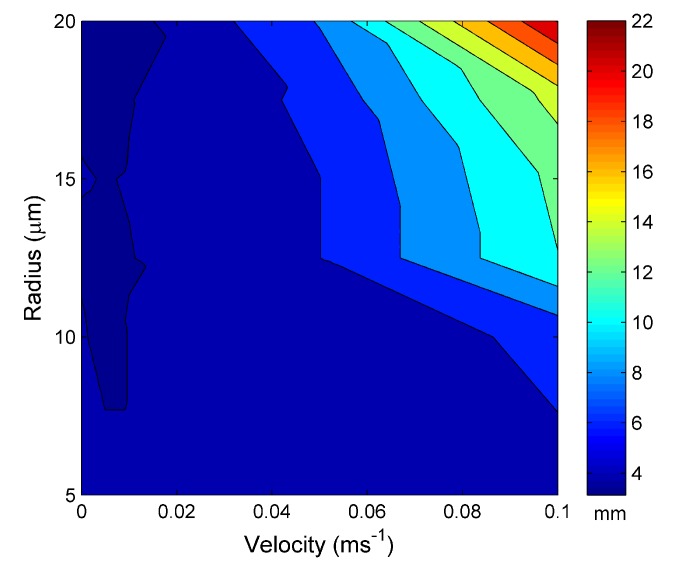
The distance taken to reach the steady-state length *vs.* the water hole radius and water input velocity at 60 °C.

Figure 7 is the distance required for the water channel to reach steady state for an external temperature of a source set at 60 °C. Figure 7 shows the same trend as Figure 5 with increasing steady-state length with increasing water channel radius and velocity. However, the maximum steady-state distance is 22 µm, 2 µm smaller than for an external temperature of 30 °C.

**Figure 8:**
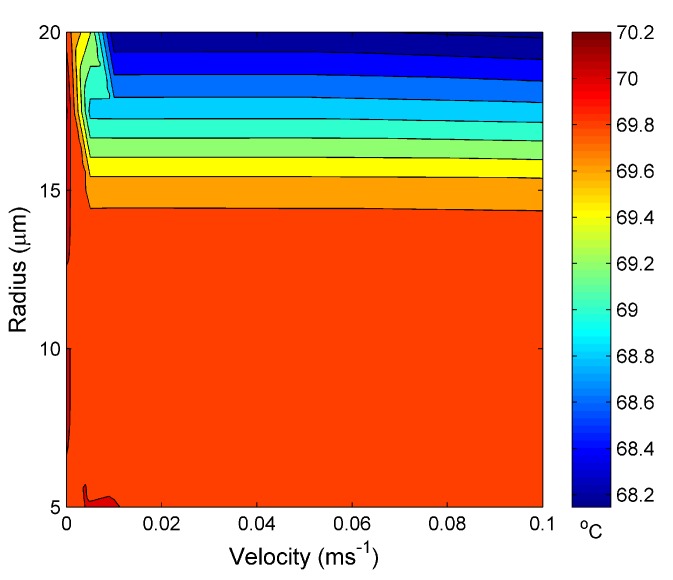
Calculated temperature at the steady state *vs.* the water hole radius and water input velocity at 60 °C.

Figure 8 is the temperature reached at the steady state for the water channel for an external temperature set at 60 °C. The trend seen in Figure 6 is, again, seen in Figure 8, except with a smaller critical channel radius of ~14 µm and a larger temperature range of ~2 °C, as would be expected from increasing the external temperature.

**Figure 9:**
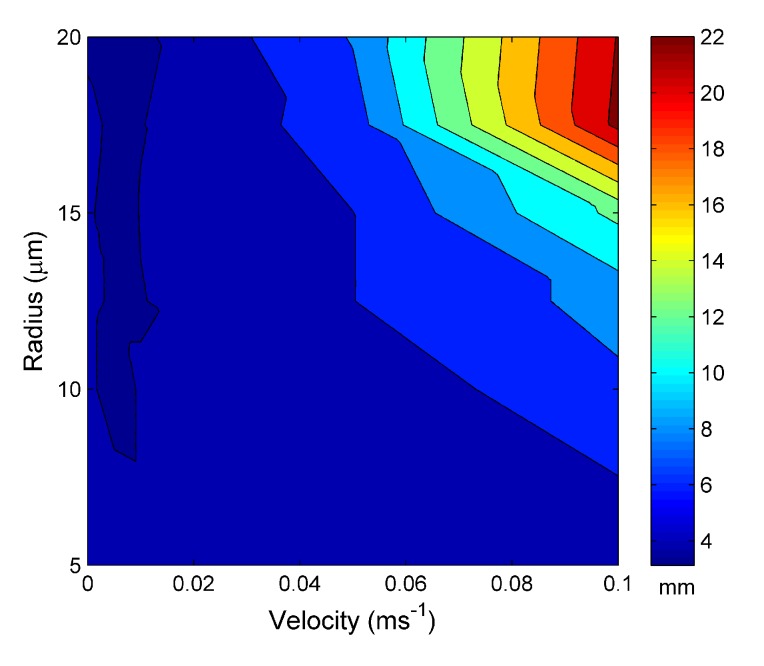
The distance taken to reach steady-state length *vs.* the water hole radius and water input velocity at 90 °C.

Figure 9 is the distance required for the water channel to reach steady state for a an external temperature of a source set at 90 °C. Again, the same general behavior is seen, with a few exceptions. Unexpectedly, the maximum length to reach equilibrium is approximately the same as that for an external temperature of 60 °C, and the reason for this will be investigated in the future. However, the data also show that at larger channel radii, above 17 µm, the behavior is dominated by velocity and is approximately independent of the channel radius.

**Figure 10:**
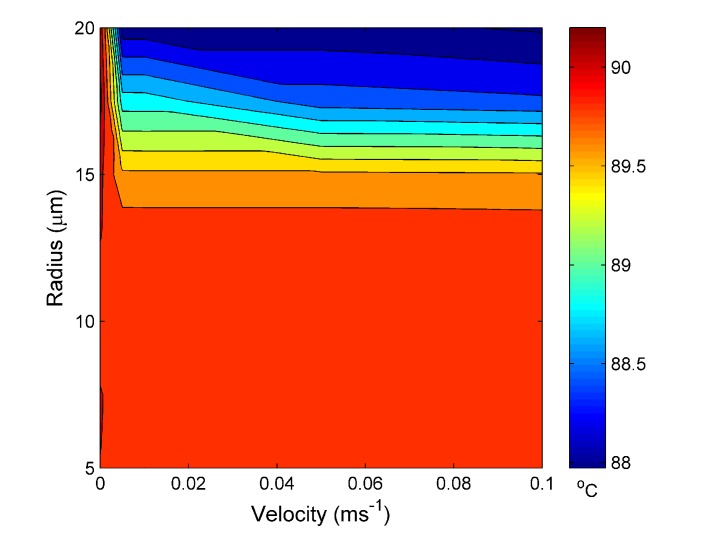
Calculated temperature at the steady state *vs.* the water hole radius and water input velocity at 90 °C.

Figure 10 shows the temperature reached at the steady state for the water channel for an external temperature set at 90 °C. Again, the magnitude of the distribution in temperature is ~2 °C, similar to the result obtained for an external temperature of 60 °C, while the critical channel radius has only slightly decreased to ~13 µm. The 60 °C and 90 °C data suggest the MOF has reached a saturation point, whereby increasing the external temperature has a decreased effect on the temperature distribution within the MOF and the distance required to reach steady state. In future work, it will be necessary to conduct an investigation to establish the relationship between the difference in *T_ext_* and the input water channel temperature and how this effects the critical channel radius.

### 4.2. Optical Modeling

**Figure 11:**
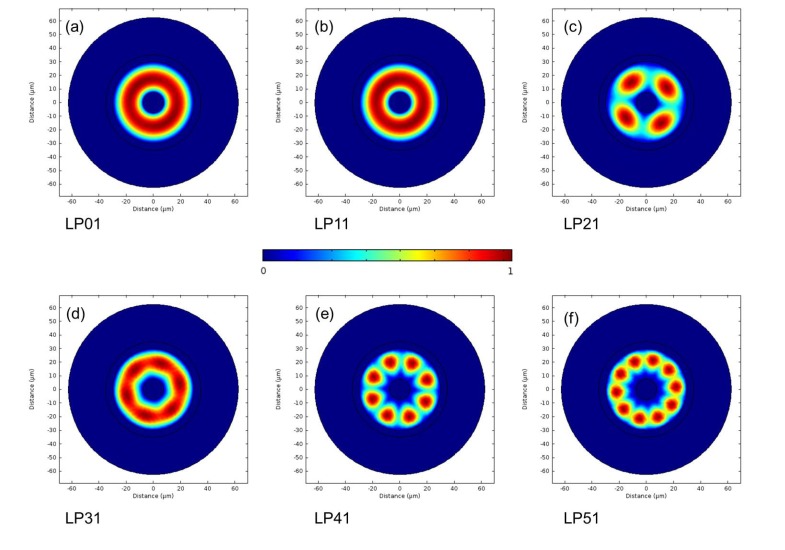
The normalized *x*-*y* plane electric field distribution for the first six calculated modes for the inner glass ring. Note: though LP01 and LP11 look similar, they are, in fact, different modes.

The normalized (*x*,*y*) electric field distributions for the first six modes of the MOF are shown in Figure 11. The modes have been labeled, similar to that of a linearly polarised (LP) mode in standard optical fiber. The first designation represents the axial dependence, while the second number describes the radial dependence; in both cases, the number represents the number of maximums for their given dependence. The electric field distribution shows that the air and water regions’ difference in the refractive index is effective at confining the modes within the core glass region.

**Figure 12:**
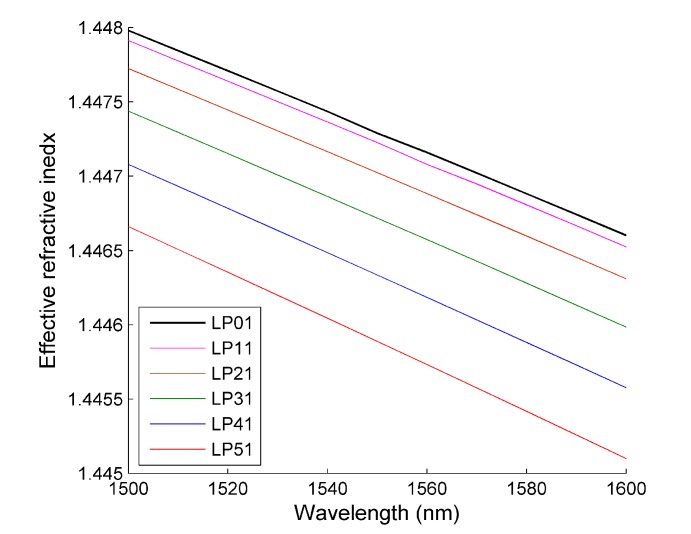
The calculated dispersion curve for the first six core modes of the optical fiber.

The simulated dispersion relationship for the modes in Figure 11 is shown in Figure 12 and shows the characteristic behavior expected for optical fiber modes. All of the modes have an effective refractive index that is between the refractive index of glass and water. For each mode, there is a decrease in the effective refractive index with increasing wavelength, which is attributed to the dispersion properties of glass and water. Finally, the difference in the effective refractive index between each mode increases with increasing mode order. This is typical behavior and is a result of an increase in the electric field outside the core glass region.

**Figure 13:**
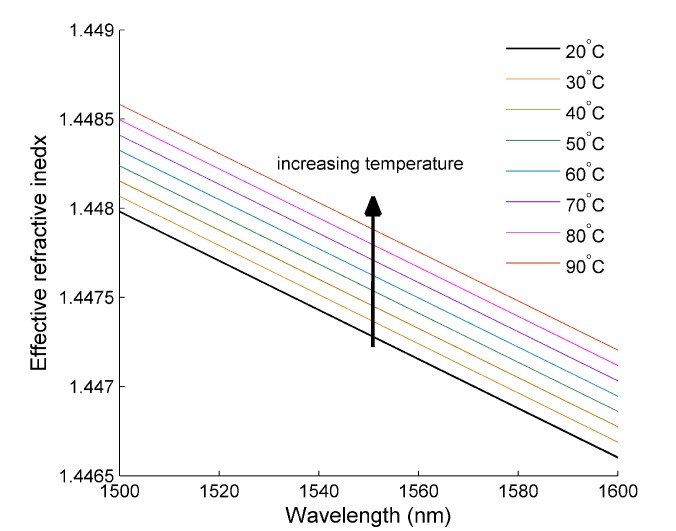
The calculated dispersion curve for the LP01 mode and how its effective index changes with respect to increasing temperature.

**Figure 14:**
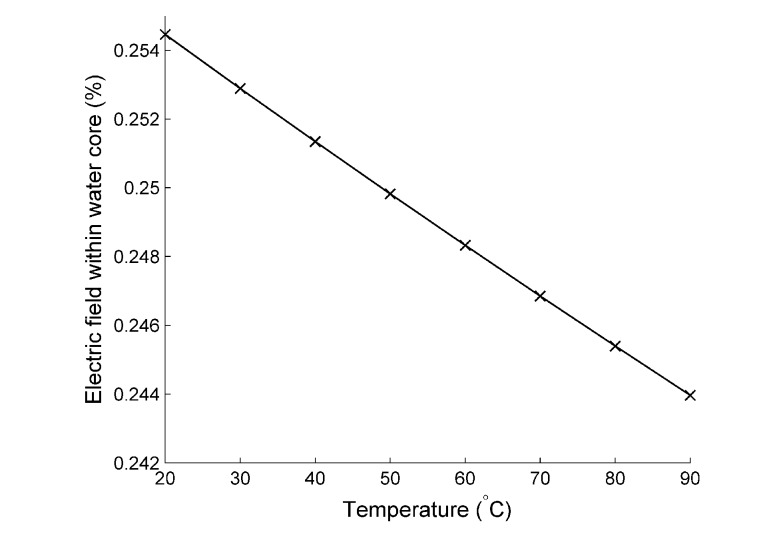
The percentage of the mode overlap within the water channel in relationto temperature

Figure 13 shows the response of the LP01 mode effective index and how it changes with respect to increasing temperature. This behavior was investigated for all of the modes; however, the response was the same in all cases, and so, this mode is chosen as a typical example. Figure 14 shows that increasing the temperature of the MOF results in an increase in the effective index, measured as 8.6 *RIU/*°C (where *RIU* is refractive index units), and is the thermo-optic coefficient of glass. At first, this may seem counter-intuitive, due to the thermo-optic coefficient of water being an order of magnitude larger and negative in sign. However, this is explained by two factors: firstly, the majority of the light is confined within the glass, which means that the influence of the thermo-optic change of water on the effective index is negligible, despite its large value; secondly, as the temperature increases further, so does the percentage of light confined within the glass region, further negating the influence of water. This is a direct effect of the difference in sign between the therm-optic coefficient of water and glass. This behavior is confirmed in Figure 13, where the percentage of the electric field within the water channel is plotted *vs.* temperature. Less than 1% of the mode can be found within the water channel, and this steadily decreases with increasing temperature.

## 5. Conclusions

In this work, we have recognized the importance of understanding the microfluidic behavior of MOFs. The importance of MOF infiltration has made such a study extremely important, with relevance to optical fiber micro-reactors, non-linear fibers and fiber lasers. We have utilized an FEA model that has been constructed to predict the thermo-fluidic and optical properties of an MOF accounting for changes in external temperature, input water velocity and optical fiber geometry. Our approach is flexible and can be readily applied to any cross-sectional fiber geometry. As we also account for wave guidance, we are able to consider the effect of the fluid channel on light guidance and the impact this has on the transmission behavior of the fiber. The results show that the steady-state temperature is dependent on the water channel radius, while independent of the input fluid velocity, except at very low velocities. There is a critical channel radius, below which the steady-state temperature of the water channel is constant, while above, the temperature decreases. However, the distance required to reach steady state within the water channel is dependent on both the input velocity and the channel radius. The MOF has been found capable of supporting multiple guided modes. Despite the large thermo-optic coefficient of water, the bound optical modes’ response to temperature was dominated by the thermo-optic coefficient of glass. This is attributed to the majority of the light being confined within the glass, which increased with increasing external temperature due, to a larger difference in the refractive index between the glass core and the water channel. Further studies are under way to account for the different host materials and fluid properties.
